# Méningiome intracrânien multiple: expérience du service de neurochirurgie CHU Avicenne Rabat - Salé, à propos de 4 cas et revue de la literature

**DOI:** 10.11604/pamj.2014.18.204.4811

**Published:** 2014-07-06

**Authors:** Ben Ousmanou Djoubairou, Claire Karekezi, Nabil Moussé, Agbéko Komlan Doleagbenou, Rachid Gana, Najia El Abbadi, Moulay Rachid El Maaqili

**Affiliations:** 1 Service de neurochirurgie CHU Avicenne, Rabat-Salé, Université Mohammed V Souissi, Rabat, Maroc

**Keywords:** Méningiome multiple, exérèse chirurgicale, neurofibromatose, multiple meningioma, surgical excision, neurofibromatosis

## Abstract

Les méningiomes intracrâniennes multiples sont définies comme la présence d'au moins deux méningiomes sur des sites intracrâniens différents et ceci en absence de neurofibromatose. C'est une tumeur rare dont la prévalence varie entre 1-10%. Le but de notre travail était de décrire les caractéristiques cliniques, radiologiques, histologiques d'une série de 4 patients porteurs de méningiome multiple et en déduire les facteurs de risques de survenue de cette pathologie. Préciser la qualité d'exérèse chirurgicale de la lésion selon la classification de Simpson. Rapporter les suites postopératoires ainsi que le suivie à long termes des patients afin de préciser leur qualité de vie. Il s'agit d'une étude rétrospective portant sur 4 cas de Méningiomes intracrâniens multiples sur 174 patients opérés pour méningiome au CHU Avicenne entre Janvier 2000 à Décembre 2013. En s'aidant des données cliniques, imageries, chirurgicales, histologiques mentionnée dans le dossier médical de chaque patient. Notre série est constitué de 4 patients (3 femmes pour 1 homme), d'un âge allant de 42-50 ans (moyenne d’âge= 45,5 ans). Nous avons identifié 21 méningiomes (17 en sus tentoriel et 4 en sous tentoriel), aucun cas de décès ni d'infection postopératoire dans notre échantillon. Le pronostic reste bon malgré le nombre de lésion nécessitant parfois plusieurs interventions chirurgicales.

## Introduction

Les méningiomes sont des tumeurs extra- axiale classiquement bénignes, développée à partir des cellules méningothéliales de l'arachnoïde et correspond à 13-20% de toutes les tumeurs intracrâniennes [1, 2]. Les méningiomes intracrâniens multiples sont caractérisés par la présence d'au moins deux méningiomes sur des sites intracrâniens différents chez un patient en dehors du cadre de neurofibromatose [2–4]. Notre étude a pour but de décrire les caractéristiques cliniques, radiologiques, histologiques d'une série de 4 patients porteurs de méningiome multiple et en déduire les facteurs de risques de survenue de cette pathologie. Préciser la qualité d'exérèse chirurgicale de la lésion selon la classification de Simpson et rapporter les suites postopératoires ainsi que le suivie à long termes des patients afin de préciser leur qualité de vie.

## Méthodes

Il s'agit d'une étude rétrospective portant sur 174 méningiomes opéré dans le service de Neurochirurgie CHU Avicenne entre Janvier 2000 et Décembre 2013. Le diagnostic a été posé sur des données cliniques, de l'imagerie cérébrale (TDM et IRM) et confirmé par l’étude anatomopathologique de la pièce opératoire après exérèse microchirurgicale. Pour chaque patient nous avons recueillis les données épidémiologiques, cliniques, imagerie, ainsi que le nombre d'interventions chirurgicales que nous avons introduit dans le Tableau 1.

**Tableau 1:** Profil démographique, clinique, histologique, nombre d'intervention, qualité d'exérèse

Cas	Age/Sexe	Localisation	Nombre	Radio pré-op	Radio post-op	Nombre chirurgie	Histologie
1	43/F	Pariétale, sphénoïdal, falcorielle	3 ST	non	oui	2	Méningiome grade 1 OMS (transitionnel)
2	47/F	Fosse postérieure, convexité frontale, falcorielle	10 ST 4 FCP	non	oui	1	Méningiome grade 1 OMS (méningothélial)
3	42/F	Frontale, pariétale	2 ST	non	non	2	Méningiome grade 1 OMS
4	50/M	Frontale	2 ST	non	non	1	Méningiome grade 1 OMS

ST : sus tentoriel, FCP : fosse cérébrale postérieure, Radio : Radiothérapie

## Résultats

Le délai de suivi de nos patients allait de 6 mois à 8 ans (moyenne= 2.6 ans). Notre échantillon était constitué de 4 patients (3 Femmes et 1 Homme) d'un âge situé entre 42-50 ans. Vingt- un méningiomes de sièges différents ont été retrouvés, 4 au niveau de la fosse cérébrale postérieure et 17 en sus tentoriel (pariétal, frontal, parasagital, suprasellaire). Le nombre de méningiome par patient varie de 2-14. Nos patients avaient bénéficiés d'une préparation médicamenteuse avant la chirurgie à base de corticothérapie et antiépileptiques, la chirurgie était planifié grâce aux données clinique et de l'imagerie. Nous n'avons pas eu des cas de décès postopératoire ni d'infection. Un cas de récidive tumoral sur le site opératoire a été retrouvé après 8 ans de suivie ce qui a motivé une deuxième intervention avec exérèse type Simpson I.

### Observation 1

**Figure 1:**
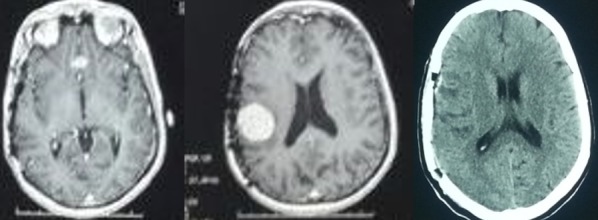
Imagerie pré et post opératoire de l'observation 1. IRM coupes axiales T1 injecté objectivant 2 méningiomes l'un au niveau du tubercule de la selle et l'autre pariétale droite; T DM c+ coupe axiale post- opératoire exérèse totale

Ancienne patiente du service âgée de 43 ans opéré il y a 8 ans pour méningiomes intracrâniens multiples (pariétale droite et suprasellaire) révélé par une épilepsie secondaire sans déficit neurologique. Elle a bénéficié d'une exérèse chirurgicale de la lésion pariétale type Simpson I avec bonne évolution post opératoire. Patiente régulièrement suivie en consultation, le dernier bilan radiologique de suivie a révélé une récidive tumoral avec aspect stationnaire du méningiome suprasellaire. Elle a bénéficié d'une deuxième intervention chirurgicale avec une exérèse macroscopiquement totale et l’étude anatomopathologique était en faveur d'un méningiome transitionnel grade I OMS identique à la précédente (Figure 1).

### Observation 2

**Figure 2:**
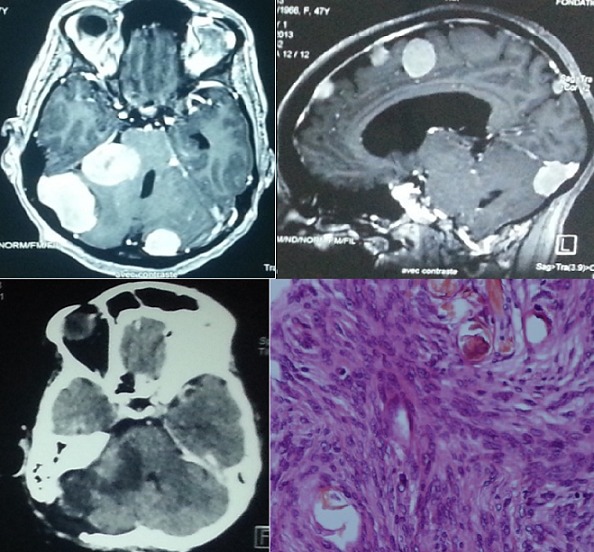
Imagerie pré et post opératoire de l'observation 2. IRM coupe Axiale et sagittale T1 injecté plusieurs méningiomes sous et sus tentorielle. TDM C+ coupe axiale exérèse totale du méningiome pétreux droit. Anatomopathologie méningiome grade 1 OMS

Patiente de 47 ans antécédents de myome utérin admise pour syndrome cérébelleux statokinétique. IRM cérébrale a objectivé 14 méningiomes (10 en supra tentoriel et 4 dans la fosse cérébrale postérieure) dont les plus volumineuses (deux) ce retrouve au niveau de la tente du cervelet droit. L'indication d'un abord direct a été retenue et elle a bénéficié d'une exérèse Simpson I pour la lésion externe et Simpson II pour la lésion affleurant le tronc cérébrale. L’étude histologique en faveur d'un méningiome méningothélial grade I OMS. Les suites opératoires étaient marquées par une paralysie faciale grade I House-Brackmann et un trouble de la déglutition ayant régressés à sa sortie à J+10 postopératoires (Figure 2).

## Discussion

**Série**: Les méningiomes multiples ont été décrit pour la première fois par Anfirmow et Blumenna [5], il est important de faire la différence entre les méningiomatoses liées à la neurofibromatose (NF) et méningiomes multiples en absence de cas familial [1, 2, 5]. La plus grande série retrouvé jusqu'ici comporte 7 patients, c'est une étude multicentrique sur une période de 10 ans (1996 - 2006) incluant deux hôpitaux brésiliens de référence; L’âge moyen des patients de cette série était de 53,8 ans avec une prédominance féminine [3]. La prévalence des méningiomes multiples dans notre série était de 2,3% comparable à ce qui a été rapporté par différent auteurs [3, 4, 6–8].

**Génétique**: La délétion du chromosome 22 dans les NF de type 2 est souvent lié à l'apparition des méningiomes multiples. D'autres parts les facteurs comme la radiothérapie utilisée pour plusieurs pathologies bénignes comme la teigne [9].

**Progestérone**: Cette théorie hormonale avancée par plusieurs auteurs fait allusion à la progestérone surtout pendant la phase lutéale du cycle menstruel et la grossesse, prédisposant ainsi la femme [10], dans notre expérience 3F/1H.

**Localisation**: La localisation la plus fréquente est en sus tentorielle, cependant il existe des cas de méningiomatose de la fosse cérébrale postérieure [3].

**Traitement**: Histoire naturelle des méningiomes multiples est semblable à celle des méningiomes, La courbe de l’évolution en termes de volume des méningiomes multiples ne parait pas supérieure à celle des méningiomes isolés, ce qui suggère un contrôle clinique et radiologique pour les lésions asymptomatiques [8]. Pour plusieurs auteurs la chirurgie n'est indiquée que sur les lésions symptomatiques, le reste devrait bénéficier d'un suivie clinique et imagerie tout les 6 à 12 mois. Pour les lésions géographiquement proche ont peut envisager un abord large permettant d'avoir accès aux deux lésions, c'est ce qui a été réalisé pour le patient n°2. Parlant de la qualité d'exérèse, elle doit autant que faire ce peut être totale (Simpson I), elle permet de retarder les récidives [11]. Le diagnostic de confirmation reste anatomopathologique, il est a noté que dans la majorité des cas on retrouve les méningiomes de grade I OMS suggérant ainsi un meilleur pronostic malgré le nombre élevé de tumeurs. Il a été rapporté des cas de méningiomes multiples de grade différents chez le même patient [12].

## Conclusion

Les méningiomes multiples en dehors de la NF sont rares et la prise en charge dépend de la localisation, volume, nombres et enfin de la symptomalogie révélant la maladie. La femme est la plus atteinte selon la théorie hormonale. Le pronostic reste bon malgré le nombre de lésion nécessitant très souvent de multiples interventions chirurgicales.
